# Lifespan‐Extending Endogenous Metabolites

**DOI:** 10.1111/acel.70371

**Published:** 2026-01-12

**Authors:** Yizhou Jiang, Jing‐Dong J. Han

**Affiliations:** ^1^ Key Laboratory of Reproductive Health Diseases Research and Translation of Ministry of Education, International Center for Aging and Cancer Hainan Medical University Haikou China; ^2^ Hainan Academy of Medical Sciences Hainan Medical University Haikou China; ^3^ Peking‐Tsinghua Center for Life Sciences, Academy for Advanced Interdisciplinary Studies, Center for Quantitative Biology (CQB) Peking University Beijing China; ^4^ Peking University Chengdu Academy for Advanced Interdisciplinary Biotechnologies Chengdu China

**Keywords:** aging, anti‐aging, lifespan, metabolism, metabolites

## Abstract

Aging is a multifactorial process influenced by genetic, environmental, and metabolic factors. Dysregulated nutrient sensing and metabolic dysfunction are hallmarks of aging, and reduction of insulin/IGF‐1 signaling or metabolic interventions such as caloric restriction extend lifespan across species. Endogenous metabolites reflect and mediate these metabolic cues, linking nutrient status to epigenetic and transcriptional programs by serving as cofactors for chromatin‐modifying enzymes or as allosteric modulators of transcription factors. Some metabolites have emerged as key regulators of longevity, integrating into networks to concurrently influence multiple aging‐related pathways. In this review, we summarize evidence supporting the lifespan‐extending effects of key endogenous metabolites across diverse model organisms and discuss their mechanisms of action. These insights underscore the potential of targeting metabolic networks as a multifaceted strategy to delay aging. Finally, we consider the translational promise of metabolite‐based interventions to extend healthspan while minimizing adverse effects, and we note remaining challenges such as optimal dosing, context‐specific effects, and demonstrating efficacy in humans.

## Graphical abstract

Endogenous metabolites act as mediators of longevity by modulating conserved cellular pathways. We summarize mechanistic evidence linking specific metabolites to lifespan and healthspan benefits across model systems, with discussion of clinical evidence, translational opportunities, and remaining knowledge gaps.

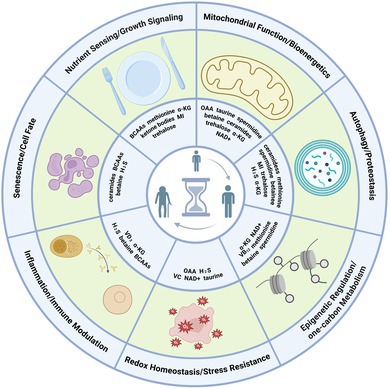

## 1 Introduction

Aging is a multifactorial process driven by the interplay of genetic, environmental, and metabolic perturbations, with dysregulated nutrient sensing and metabolic dysfunction recognized as core hallmarks (Lopez‐Otin et al. 2023). Interventions that alter metabolite availability or metabolism, such as caloric restriction (CR) or reduced insulin/IGF‐1 signaling (IIS), extend lifespan across species (Gao et al. 2018). Metabolites, the small‐molecule intermediates and end products of cellular pathways, both reflect and mediate the integrated response to genetic and environmental cues (Adav and Wang 2021). They act as cofactors for chromatin‐modifying enzymes and bind or allosterically regulate gene regulatory proteins, thereby directly linking cellular metabolic state to epigenetic and transcriptional programs. Through these metabolite‐derived cues, nutritional and environmental inputs are translated into context‐dependent changes in gene expression that can influence physiological and pathological processes (Hornisch and Piazza 2025; Meier 2013). Consequently, some endogenously produced metabolites have emerged as pivotal modulators of longevity. Their advantages as geroprotectors are multifaceted. Unlike synthetic compounds that target single pathways, metabolites integrate naturally into biological systems and modulate multiple aging mechanisms like IIS, sirtuin activity, autophagy, and redox homeostasis, thereby addressing the complexity of aging in an integrated manner. Moreover, metabolites are often biocompatible as they are endogenous or dietary, reducing toxicity and enhancing translational potential. This review highlights the role of endogenous metabolites in regulating longevity pathways and evaluates evidence across species to aid understanding metabolic regulation of aging and guiding future investigations. By linking fundamental aging biology to clinical applications, metabolites enable interventions that extend healthspan with minimal adverse effects, highlighting their key role in geroscience.

## 2 Physiological Functions of Endogenous Metabolites

Endogenous metabolites are small molecules produced by an organism's own metabolism. They encompass a wide range of molecules, such as amino acids, lipids, nucleotides, and sugars, which are pivotal for cellular function and organismal health (Baker and Rutter 2023). Beyond serving as biosynthetic precursors and energy substrates, many metabolites also function as dynamic modulators of signaling and gene regulatory networks by engaging in protein–metabolite interactions, allosteric regulation, and by serving as substrates for chromatin and other post‐translational modifications (Boon et al. 2020; Hornisch and Piazza 2025). Metabolites can function as extracellular signals activating G protein‐coupled receptors (GPCRs), such as free fatty acid receptors for fatty acids, GPR81 for lactate, SUCNR1 for succinate, and TGR5 for bile acids (Tonack et al. 2013). These GPCRs are expressed in gut, adipose tissue, endocrine glands, and immune cells, linking nutrient and metabolite levels to diverse physiological responses (Tonack et al. 2013). Other metabolites serve as enzyme cofactors or epigenetic regulators. For example, methyl donors like betaine provide methyl groups for DNA and histone methylation and also act as osmolytes to protect cells under stress (Lever and Slow 2010). Some metabolites even form specialized structural assemblies. For instance, guanine crystals can form structural color in feline eyes and contribute to enhanced night vision (Aizen et al. 2018).

Perturbations of endogenous metabolite levels or fluxes have been linked to genomic instability, metabolic dysfunction, and age‐related diseases, motivating study of metabolites as both biomarkers and functional modulators of aging (Adav and Wang 2021; Tomar and Erber 2023; Xiao et al. 2025). Metabolomic studies reveal characteristic metabolite changes in diabetes, cardiovascular disease, and Alzheimer's disease (AD) (Panyard et al. 2022), suggesting that metabolites not only reflect organismal state but also can actively influence aging pathways. In subsequent sections, we will examine specific endogenous metabolites implicated in longevity regulation.

## 3 Taurine

Taurine is a sulfur‐containing β‐amino acid synthesized endogenously from cysteine or methionine and present at high concentrations in many mammalian tissues such as heart, brain, retina, and skeletal muscle (Jong et al. 2021; Wu 2020). It is not incorporated into proteins but functions in osmoregulation, calcium homeostasis, and neurotransmission (De Luca et al. 2001; El Idrissi et al. 2013; Jong et al. 2021; Liu et al. 2025). Taurine has also been implicated in antioxidant and anti‐inflammatory defenses, partly by supporting mitochondrial protein synthesis and function (Jong et al. 2021).

Taurine supplementation shows protective effects in aging models. In UVB‐irradiated hairless mice, oral taurine preserved epidermal moisture and significantly reduced wrinkle formation, suggesting an anti‐photoaging action via its osmolyte and moisturizing roles (Yoshimura et al. 2021). In aged mice, taurine significantly ameliorated the age‐dependent decline in spatial memory (El Idrissi et al. 2013). Taurine‐treated aged mice also had higher hippocampal levels of GABA and glutamate, increased expression of both isoforms of glutamate decarboxylase (GAD) 65/67 and of somatostatin, and larger CA1 population spikes with enhanced paired‐pulse facilitation (El Idrissi et al. 2013). These taurine‐induced changes in the inhibitory system oppose those normally observed in the aging brain.

Taurine has recently been proposed as a circulating factor linked to aging, yet another comprehensive analysis has reported substantial interindividual variability in circulating taurine levels. Singh et al. measured taurine concentrations in mice, monkeys, and humans and reported an age‐related decrease, and they found that reversing this decline by taurine supplementation increased healthspan and lifespan in worms and rodents (Singh et al. 2023). It also reduced cellular senescence, DNA damage, mitochondrial dysfunction, and inflammaging. Singh et al., therefore, proposed that taurine deficiency could be a driver of aspects of aging, and they recommended clinical trials in humans to test this hypothesis. In contrast, Fernandez et al. analyzed longitudinal and cross‐sectional data from multiple cohorts and observed that circulating taurine concentrations increased or remained unchanged with age and that associations between taurine and age‐related health outcomes were inconsistent across populations (Fernandez et al. 2025). Fernandez et al., therefore, concluded that low circulating taurine is unlikely to serve as a universal biomarker of aging and emphasized that taurine effects are likely context dependent (Fernandez et al. 2025). A recent study in humans that measured circulating taurine in 137 men similarly found no association between taurine and either age or physical performance (Vincent Marcangeli et al. 2025). The authors also concluded that circulating taurine does not appear to track human biological aging or functional decline in their sample, though they note that this does not exclude potential benefits from supplementation in specific subgroups (Vincent Marcangeli et al. 2025). Taken together, these findings indicate that, although taurine can modulate cellular resilience and mitochondrial parameters in experimental models, its value as a systemic biomarker of natural aging is uncertain and likely depends on species, population, and physiological context. Resolving these discrepancies will require well‐powered longitudinal studies, tissue‐specific measurements, standardized assays, and randomized supplementation trials to determine whether taurine supplementation benefits defined human subpopulations.

Taken together, taurine is an endogenous osmolyte and antioxidant that supports mitochondrial and neural health. Animal studies suggest that supplementation can mitigate age‐related deficits in cognition, cellular senescence, and tissue function (El Idrissi et al. 2013; Singh et al. 2023). Evidence on natural taurine changes during healthy aging is mixed, highlighting species and individual variability (Fernandez et al. 2025; Singh et al. 2023; Vincent Marcangeli et al. 2025). Further studies should clarify taurine metabolism in aging and identify human populations that could benefit from supplementation without adverse effects.

## 4 Betaine

Betaine, also called trimethylglycine, is a naturally occurring trimethylated amino acid present in plants, animals, and humans. It is endogenously synthesized from choline and also obtained from the diet (Arumugam et al. 2021). In the body, it primarily functions as an osmolyte and as a methyl‐group donor in one‐carbon metabolism (Arumugam et al. 2021). Betaine donates a methyl to homocysteine via betaine–homocysteine methyltransferase (BHMT) to regenerate methionine and S‐adenosylmethionine (SAM), increasing the cellular SAM: SAH (S‐adenosylhomocysteine) ratio (Craig 2004; Dai et al. 2022; Zawieja and Chmurzynska 2025).

Emerging evidence across model organisms indicates that betaine can delay the aspects of aging. In *Caenorhabditis elegans* ( *C. elegans* ), three anti‐aging compounds—metformin, quercetin and minocycline—raised endogenous betaine levels and direct betaine supplementation prolonged worm lifespan, indicating that betaine is a key age‐associated metabolite (Lan et al. 2024). This pro‐longevity effect involves conserved stress‐response mechanisms, including increased DAF‐16/FOXO (the forkhead box transcription factor class O) and p38‐MAPK signaling, enhanced autophagy by mTOR inhibition, and reduced oxidative stress (Lan et al. 2024).

In aged mice, dietary betaine improved skeletal muscle mass, strength, and endurance, with preserved mitochondrial structure and respiration (Chen, He, et al. 2024). Mechanistically, betaine upregulated the transcription factor Yin Yang 1, which repressed the novel mitochondrial regulator Mss51, thereby maintaining the expression of mitochondrial electron‐transport genes and respiration (Chen, He, et al. 2024). Betaine also enhanced autophagy in aging muscle via a SAM‐dependent mechanism, increasing SAM levels to stimulate the methyltransferase Mettl21c, which trimethylates the p97 and drives autophagic turnover (Chen, Chen, et al. 2024). Consequently, betaine‐treated animals show higher levels of autophagy markers (Atg5, Atg7, LC3‐II, Beclin1), increased autophagic flux, and ameliorated age‐related muscle loss and functional decline (Chen, Chen, et al. 2024). Betaine supplementation also improved cognition in a rat model of hyperhomocysteinaemia, reducing ROS and inhibiting homocysteine‐induced microglial activation and pyroptosis (Yang et al. 2024). Notably, betaine increased the SAM/SAH ratio and enhanced m^6^A methylation on NLRP3 mRNA, recruiting the YTHDF2 reader to destabilize NLRP3 transcripts and thereby blunt the NLRP3/caspase‐1/GSDMD pyroptosis pathway (Yang et al. 2024).

At a systemic level, a recent study indicates that exercise elevates circulating betaine, and betaine by itself mimics some exercise‐induced benefits (Geng et al. 2025). Multi‐omics revealed that repeated exercise increased circulating betaine in humans, and betaine supplementation ameliorated age‐related decline in multiple tissues in mice. Mechanistically, betaine directly binds to and inhibits TANK‐binding kinase 1 (TBK1), suppressing TBK1‐dependent inflammatory signaling, reducing markers of cellular senescence and inflammation, and improving physiological outcomes in aged animals (Geng et al. 2025). These results position betaine as an exercise‐induced humoral factor that links metabolic changes to innate immune signaling and downstream geroprotective effects.

Despite these promising findings, important uncertainties remain. For example, although plausible mechanisms exist whereby betaine could indirectly reduce cardiovascular disease and diabetes risk, some large prospective studies and meta‐analyses do not consistently link dietary betaine to reduced disease incidence (Bidulescu et al. 2007; Dibaba et al. 2020; Meyer and Shea 2017; Zawieja and Chmurzynska 2025). In the Atherosclerosis Risk in Communities (ARIC) cohort, higher dietary choline was associated with an increased risk of type 2 diabetes in women but not in men (Dibaba et al. 2020). This sex‐specific association is plausibly explained by sex hormone‐driven differences in hepatic one‐carbon enzymes that result in higher choline and betaine metabolites in females (Sadre‐Marandi et al. 2018). Trimethylamine‐N‐oxide (TMAO) is associated with increased cardiovascular risk, and both dietary choline and betaine can be metabolized via gut microbial trimethylamine into TMAO, although betaine is a weaker precursor than choline (Wang et al. 2011; Yu et al. 2020). Therefore, betaine may raise TMAO and thereby confound epidemiological associations between dietary betaine and cardiometabolic outcomes. In addition, high‐dose betaine (4–6 g/day) has been associated with increased total and low‐density lipoprotein (LDL) cholesterol in some trials and meta‐analyses, posing a safety concern for obese or prediabetic individuals (Ashtary‐Larky et al. 2022; Olthof et al. 2005). Translational evidence and optimal human dosing for the anti‐aging effect of betaine remain limited, warranting controlled clinical trials.

## 5 α‐Ketoglutarate

α‐Ketoglutarate (α‐KG) is a central tricarboxylic acid (TCA) cycle intermediate produced from isocitrate by isocitrate dehydrogenase and from glutamate via transamination or dehydrogenation (Chmelova et al. 2024; Plaitakis et al. 2017; Wu et al. 2024). It is converted by the α‐KG dehydrogenase complex to succinyl‐CoA (Wu et al. 2024). In *C. elegans* , α‐KG supplementation at 8 mM during adulthood extends lifespan by approximately 50% and delays age‐related functional decline (Chin et al. 2014). Mechanistically, α‐KG directly binds to and inhibits the beta subunit of ATP synthase (ATP5B/ATP‐2), as identified through Drug Affinity Responsive Target Stability (DARTS) profiling. This inhibition reduces cellular ATP levels and oxygen consumption while activating autophagy in both worms and mammalian cells. Similarly, inhibition of ATP synthase by oligomycin or RNAi of *atp‐2* also extends worm lifespan, mimicking the effect of α‐KG. α‐KG extends lifespan through a dietary restriction (DR)‐like mechanism, as it fails to further extend longevity in *eat‐2* mutants, a genetic model of DR. Within classical aging‐regulatory mechanisms, α‐KG lifespan extension requires TOR signaling, is partially dependent on the AAK‐2/AMPK (AMP‐activated protein kinase)‐DAF‐16/FOXO signaling pathway, yet independent of insulin/IGF‐1 receptor DAF‐2 and HIF‐1 (Chin et al. 2014). Physiologically, endogenous α‐KG levels increase during starvation in *C. elegans* , and its exogenous supplementation cannot augment longevity under DR, positioning α‐KG as a key metabolite mediating the pro‐longevity effects of nutrient limitation through ATP synthase inhibition and subsequent TOR pathway modulation (Chin et al. 2014).

The anti‐aging effects of α‐KG were further validated in other model organisms. In *Drosophila*, dietary supplementation of 5 μM α‐KG extended their lifespan but reduced fecundity. α‐KG supplementation activates the AMPK signaling pathway but inhibits the mTOR signaling pathway, consistent with findings in *C. elegans* (Su et al. 2019). In mice, α‐KG extended both the lifespan and healthspan, associated with reduced systemic inflammation and increased IL‐10 (Asadi Shahmirzadi et al. 2020). Further studies suggested that α‐KG could ameliorate age‐related diseases or function declines in mice, such as osteoporosis, via regulating histone methylations (Wang et al. 2020), pressure overload‐induced chronic cardiac dysfunction (An et al. 2021), oocyte aging (H. Wang, Xu, Li, et al. 2023), and age‐related and surgery‐induced temporomandibular joint osteoarthritis (Ye et al. 2024).

However, clinical validation of α‐KG supplementation in humans remains preliminary. A retrospective analysis of a commercial α‐KG–containing formulation (Rejuvant) reported reduced DNA‐methylation‐based biological age over 7 months in a small cohort, but the specific contribution of α‐KG alone to the observed changes remains unknown (Demidenko et al. 2021). A randomized, placebo‐controlled trial of calcium‐α‐ketoglutarate (Ca‐AKG) has been registered and a detailed protocol published, but without published results yet (Sandalova et al. 2023). Therefore, existing human data are limited, and robust clinical validation is still required before concluding translational efficacy in humans.

## 6 Oxaloacetate

Oxaloacetate (OAA) is an endogenous four‐carbon metabolite of the citric acid cycle (Williams et al. 2009). It is produced by oxidation of malate via malate dehydrogenase and condenses with acetyl‐CoA to form citrate, linking glycolysis and TCA metabolism. In *C. elegans* , dietary OAA supplementation extends lifespan, requiring AMPK and the FOXO transcription factor DAF‐16 (Williams et al. 2009). This effect was hypothesized to result from OAA conversion to malate, consuming NADH and raising the NAD^+^/NADH ratio to mimic CR (Williams et al. 2009). Consistent with an evolutionarily conserved role, comparative metabolomics across 11 *Drosophila* species found that OAA levels in older females co‐evolved with species lifespan, indicating that OAA‐associated metabolic modules track longevity (Harrison et al. 2022).

At the cellular level, OAA metabolism is linked to mitochondrial function. The oxaloacetate decarboxylase FAHD1 is required for electron transport chain (ETC) function, since FAHD1 deficiency, which would raise OAA, impaired ETC function and triggered premature senescence in human cells (Etemad et al. 2019). Conversely, FAHD1 overexpression, which leads to depletion of mitochondrial OAA, reduces reactive oxygen species in human cells by decreasing the flux of the TCA cycle, linking OAA flux to redox balance (Heberle et al. 2024). In worms, the FAHD1 ortholog FAHD‐1 was shown to modulate neural signaling. Loss of *fahd‐1* impaired locomotion and egg‐laying and induced enzymes of serotonin biosynthesis, highlighting a connection between OAA metabolism and neurotransmitter signaling (Baraldo et al. 2019). Loss of *fahd‐1* also resulted in decreased mitochondrial function and longevity (Taferner et al. 2015).

However, translation of findings from invertebrates to mammals has been inconsistent. According to a lifelong intervention performed by the National Institute on Aging Interventions Testing Program (ITP) using genetically heterogeneous UM‐HET3 mice, OAA administered from 4 months of age did not produce a statistically significant lifespan extension (Strong et al. 2013). OAA has also been tested in an amyotrophic lateral sclerosis (ALS) model. In SOD1^G93A^ mice, OAA treatment improved neuromuscular strength and delayed paralysis without significantly altering lifespan, while normalizing spinal cord inflammatory and metabolic markers such as TNFα, NF‐κB, and PGC‐1α, implying that its benefits may involve modulation of neuroinflammation and bioenergetic stress (Tungtur et al. 2021). In humans, a pharmacokinetic study showed that oral OAA at 100 mg twice daily produced only modest and variable elevations in plasma OAA and the authors noted that relatively high endogenous OAA complicated measurement and interpretation (Swerdlow et al. 2016). A more recent randomized safety and target‐engagement study in AD patients showed that high‐dose OAA (1000 mg twice daily for 1 month) was safe and engaged brain energy metabolism, though it did not yield cognitive improvement in the short trial period and failed to demonstrate consistent plasma OAA increases (Vidoni et al. 2021). The instability of oxaloacetate likely reduces its persistence in plasma and may contribute to insufficient tissue exposure under the tested conditions and to the absence of reproducible anti‐aging effects in mammalian studies (Pudlik and Lolkema 2011).

Taken together, endogenous OAA engages conserved longevity pathways, mitochondrial bioenergetics, redox balance, and stress responses across species. While it extends lifespan in worms, mammalian evidence does not yet demonstrate a reproducible lifespan extension in healthy animals under tested conditions. These mixed results emphasize possible species‐ or genotype‐dependent effects together with practical limits such as the chemical instability of OAA, and justify follow‐up studies that optimize formulation and dosing, improve oxaloacetate stability and tissue exposure, and assess rigorous healthspan and lifespan endpoints.

## 7 Hydrogen Sulfide

Hydrogen sulfide (H_2_S) is an endogenous gasotransmitter produced in animal cells mainly by cystathionine β‐synthase (CBS), cystathionine γ‐lyase (CSE), and 3‐mercaptopyruvate sulfurtransferase (3‐MST) (Cirino et al. 2023; Sokolov et al. 2021). H_2_S has been shown to modulate aging in organisms ranging from worms to mammals. In *C. elegans* , exposure to H_2_S induces thermotolerance and extends lifespan (Miller and Roth 2007). These effects require SIR‐2.1 but are independent of IIS, mitochondrial dysfunction, or CR (Miller and Roth 2007). Furthermore, inhibition of mTORC1 or translation activates the integrated stress response transcription factor ATF‐4, which upregulates the *C. elegans* cystathionine‐γ‐lyase ortholog *cth‐2*, thereby increasing endogenous H_2_S production. This elevation of H_2_S enhances protein cysteine persulfidation and stabilizes the proteome, which contributes to lifespan extension (Statzer et al. 2022).

H_2_S levels generally decline with age, correlating with increased oxidative stress and inflammation (Blackwood and Glembotski 2022; Testai et al. 2020). In aging mice, reduced endogenous H_2_S correlates with disrupted diurnal cardiac function and elevated ROS, whereas exogenous H_2_S treatment via H_2_S donor NaHS restores the normal day–night variation of ejection fraction and lowers oxidative stress (Zhang et al. 2021). Likewise, in aged mouse kidneys, H_2_S deficiency accompanies decreased AMPK activity and activation of the insulin receptor (IR)/IRS‐2/Akt/mTORC1 translation axis. Administration of the H_2_S donor NaHS reactivates AMPK and suppresses the IR/IRS‐2/Akt/mTORC1 signaling and decreases senescence‐associated secretory phenotype (SASP) markers (Lee et al. 2018).

In mammals, H_2_S also exerts neuroprotective effects. In AD models, the H_2_S‐producing enzyme CSE is depleted and Tau is hyperphosphorylated, whereas H_2_S restores sulfhydration of glycogen synthase kinase 3β (GSK3β) to inhibit Tau hyperphosphorylation and ameliorate cognitive deficits (Giovinazzo et al. 2021). Administration of H_2_S donor sodium GYY4137 (NaGYY) ameliorates motor and cognitive deficits of AD mice (Giovinazzo et al. 2021). Similar anti‐aging effects are seen in human tissues. For example, H_2_S levels inversely correlate with premature senescence in fetal membranes, and H_2_S donors retard epithelial cell aging and matrix metalloproteinase (MMP) expression to preserve membrane integrity (J. Wang, Xu, Chao, et al. 2023).

In summary, while H_2_S robustly extends lifespan in *C. elegans* and rodent studies report organ‐level protection and improved some age‐related dysfunctions with various H_2_S donors, evidence for H_2_S directly extending lifespan in mammals is lacking. As direct administration of gaseous H_2_S is impractical, approaches such as using chemical donors, pharmacologically boosting endogenous H_2_S production or inhibiting H_2_S degradation could be explored (Sokolov et al. 2021). Definitive translation requires controlled rodent longevity studies using well‐characterized donors, standardized H_2_S level measurements, and optimized delivery strategies before human aging trials.

## 8 Myo‐Inositol

Myo‐inositol (MI), the most common isomer of inositol, is a cyclic six‐carbon polyol that is abundant in many foods and in organs such as kidney and brain (Chhetri 2019; Clements Jr. and Darnell 1980; DiNicolantonio and O'Keefe 2022; Whiteside et al. 1991). It can be synthesized from glucose‐6‐phosphate and serves as a precursor of phosphatidylinositol and membrane phosphoinositides (Bevilacqua and Bizzarri 2018). MI plays key roles in signal transduction, energy metabolism, nucleic acid synthesis, osmoregulation, and regulation of neuronal connectivity (DiNicolantonio and O'Keefe 2022; Paquette et al. 2023; Rivera et al. 2021).

Shi et al. reported that MI extends lifespan and healthspan in *C. elegans* , and the anti‐aging effect was also conserved in mice (Shi et al. 2020). Mechanistically, MI extends the worm lifespan through the PTEN homolog *daf‐18* and PTEN‐induced kinase‐1 (*pink‐1*)‐mediated mitophagy, but independently of AKT or DAF‐16/FOXO (Shi et al. 2020). Yang et al. showed that, after ruling out osmotic pressure effects, MI still extended worm lifespan and inhibited PI3K activity in a dose‐dependent manner. By contrast, MI reduced AKT phosphorylation and induced nuclear translocation of DAF‐16, and loss of AKT‐1 or DAF‐16 abolished MI's anti‐aging effect, indicating that MI's longevity effects require the AKT‐DAF‐16 pathway (Yang et al. 2023). These contrasting findings may reflect differences in experimental conditions such as osmotic stress from high MI. Importantly, both studies implicate modulation of IIS in the effects of MI, with Shi et al. supporting mitophagy mediated by PTEN and PINK‐1 and Yang et al. providing evidence for direct PI3K inhibition with downstream AKT/DAF‐16 involvement.

In mammals, emerging evidence links myo‐inositol to age‐associated functional decline. Treating middle‐aged mice with ginsenosides, an anti‐aging active component of the herb *Panax ginseng* , resulted in improved health status and attenuated DNA damage in major organs (Mingyao et al. 2024). Ginsenosides increased the liver MI content via enhanced phosphatidylcholine (PC)‐to‐MI conversion, and MI levels were positively correlated with cardiac function in senescent mice. In addition, MI attenuated aging in cardiomyocytes by reducing ROS and DNA damage (Mingyao et al. 2024). These findings highlight that the hepatic PC‐MI pathway mediates the anti‐aging effects of ginsenosides.

In the nervous system, elevated hippocampal MI has been associated with glial activation and mild cognitive impairment in aged mice (Ebert et al. 2021). Human magnetic resonance spectroscopic imaging revealed region‐specific associations of MI with sleep and cognition. Elevated MI in the bilateral frontal lobes was linked to slower processing speed and reduced sleep efficiency, while in frontoparietal regions, it correlated with better cognitive performance (Mueller et al. 2024). Myo‐inositol is highly enriched in breast milk and remains bioactive in mature brain tissue, where its supplementation promoted synapse formation in human excitatory neurons, cultured rat neurons, and the mouse cortex (Paquette et al. 2023). These findings suggest that MI supports neuronal connectivity in both developing and mature brain tissue, raising the question of whether similar mechanisms might also influence synaptic maintenance during aging, a possibility that remains to be tested. However, its links to glial activation, cognitive impairment, and divergent region‐specific associations in the aging brain raise the possibility that MI may exert both beneficial and detrimental effects depending on brain region, cell type, or stage of life. The overall impact of MI on brain aging remains to be determined. Moreover, direct evidence of MI supplementation on mammalian lifespan is lacking, and future work is needed to assess MI's role in aging in higher organisms and to establish its relevance for human aging markers.

## 9 NAD^+^

Nicotinamide adenine dinucleotide (NAD^+^) is a ubiquitous redox coenzyme which is central to cellular energy metabolism (Covarrubias et al. 2021). It also serves as a substrate or cofactor for sirtuins, PARPs, and other enzymes that regulate DNA repair, chromatin remodeling, and stress responses (Covarrubias et al. 2021). NAD^+^ levels decline with advancing age and lower NAD^+^ is correlated with a range of chronic age‐related disorders (Camacho‐Pereira et al. 2016; Schultz and Sinclair 2016; Yusri et al. 2025). Its effects on hallmarks of aging and lifespan have been extensively reviewed elsewhere (Belenky et al. 2007; Chini et al. 2024; Covarrubias et al. 2021; Verdin 2015) and will therefore not be discussed in detail here. In model organisms, boosting NAD^+^ via precursors nicotinamide riboside (NR) or nicotinamide mononucleotide (NMN) delays age‐associated physiological declines (Wang et al. 2016; Yoshino et al. 2011). Recent clinical studies also report some beneficial effects of NAD^+^ precursor supplementation. A randomized, dose‐ranging trial of oral NMN (300–900 mg/day for 60 days) in 80 healthy middle‐aged adults showed dose‐dependent increases in blood NAD, good tolerability, improvements in 6‐minute walk distance and self‐reported health, but no significant change in Homeostatic Model Assessment for Insulin Resistance (HOMA‐IR) (Yi et al. 2023). In older adults with mild cognitive impairment, a 10‐week randomized NR trial safely achieved a 2.6‐fold rise in blood NAD^+^ yet produced no improvement in cognition (Orr et al. 2024). A 4‐week open‐label NR pilot trial in eight older adults with peripheral artery disease reported preliminary improvements in peripheral endothelial function, cerebrovascular responsiveness, and cognition, although larger controlled trials are required to confirm these preliminary findings (Szarvas et al. 2025). However, important translational uncertainties remain about bioavailability, tissue specificity, and long‐term safety (Poljsak et al. 2022). Moreover, potential adverse effects of NAD^+^ elevation were reported, including glucose intolerance, upregulation of SASP, and potential pro‐tumorigenic side effects (Nacarelli et al. 2019; Poljsak et al. 2022). Therefore, efforts should focus on large, longer duration randomized trials that incorporate tissue‐level pharmacokinetics and pharmacodynamics, standardized functional endpoints, and dedicated safety surveillance, and also identify subgroups most likely to benefit or to be harmed.

## 10 Methionine

Methionine is an essential amino acid critical for protein synthesis and serves as a precursor for SAM, a major methyl donor involved in numerous methylation reactions including DNA and protein methylation (Cavuoto and Fenech 2012; Parkhitko et al. 2016). Methionine restriction (MetR) extends lifespan across diverse models. In yeast, MetR prolongs chronological lifespan by inducing autophagy (Ruckenstuhl et al. 2014). Deletion of autophagy genes (ATG5, ATG7, ATG8) abolishes the benefit, and MetR specifically enhances vacuolar acidification, which is required for MetR‐induced longevity (Ruckenstuhl et al. 2014). MetR also extends lifespan by boosting SAM synthesis and activating Snf1, the yeast homolog of AMPK (Ogawa et al. 2016), via mechanisms distinct from restriction of other nutrients like glutamic acid or glucose (Wu et al. 2013).

In *C. elegans* , feeding bacteria is the main source of methionine, although the worms can still produce some methionine themselves. Metformin was reported to extend worm lifespan by altering bacterial folate and methionine metabolism (Cabreiro et al. 2013). The lifespan extension requires the worm SAM synthase SAMS‐1 but not host methionine synthesis (METR‐1), indicating a key role for host–microbiota metabolic interaction. Similarly, glycine, a one‐carbon donor, has also been shown to extend worm lifespan in a methionine‐cycle–dependent manner, requiring METR‐1 and SAMS‐1 (Liu et al. 2019), highlighting the role of methionine metabolism and one‐carbon flux in longevity.

In *Drosophila*, early‐adult MetR (10% of normal level) extends lifespan with or without decreasing total amino acid levels, and the pro‐longevity effects are decreased or even lost when MetR is applied later in life (Kosakamoto et al. 2023). Dietary methionine has complex effects, as methionine alone boosts fecundity without shortening lifespan, while adding other essential amino acids to DR diet shortens life through interactions with methionine (Grandison et al. 2009). Notably, reduced IIS protects against the lifespan shortening of a fully supplemented diet. These findings suggest that monitoring the balance of methionine and other nutrients in the diet may offer a strategy to extend longevity without reducing fecundity. Metabolomic studies show that aging flies accumulate SAH and reprogram methionine metabolism, and tissue‐specific knockdown of noncanonical SAH hydrolases (dAhcyL1/2) and suppression of H3K4 trimethylation (H3K4me3) extends lifespan and healthspan (Parkhitko et al. 2016), implicating epigenetic effects in the methionine‐mediated effects on aging.

In mammals, MetR also recapitulates many lifespan and short‐term metabolic benefits. In mice, it reduces adiposity and body size, reverses age‐induced alterations in physical activity and glucose tolerance, and restores a younger metabolic phenotype (Ables et al. 2016; Lees et al. 2014). Reducing dietary methionine concentration from 0.86% to 0.17% increased rat lifespan by 30% (Orentreich et al. 1993). It was further reported that the ideal range of MetR in mice is 0.12%–0.25% to induce transcriptional and physiological benefits without affecting growth (Forney et al. 2017). A study on 18‐month‐old CB6F1 mice indicated that metabolic and gene‐expression changes induced by CR and MetR are quite different (Sun et al. 2009). Importantly, MetR‐induced lifespan extension appears to engage conserved nutrient‐sensing and aging‐related pathways. It requires autophagy and reduced TOR signaling in yeast (Ruckenstuhl et al. 2014). The mechanism can be different in higher organisms. For example, in *Drosophila*, MetR extends lifespan through *foxo*‐induced expression of *Methionine sulfoxide reductase A* (*MsrA*) (Kosakamoto et al. 2023). In mice, MetR did not alter mTOR activity (Sun et al. 2009), but lowers IGF‐1 and raises AMPK/FGF21 activity (Ables et al. 2016; Lees et al. 2014). These specific differences imply that further studies are needed to elucidate the molecular mechanisms by which MetR mediates anti‐aging effects in mammals. Additionally, MetR alters hepatic SAM and SAH and preserves DNA methylation in mice, and the observed correlation between SAH and DNA methylation suggests that changes in DNA methylation may contribute to lifespan extension by an MR diet and warrant further investigation (Mattocks et al. 2017).

## 11 Branched Chain Amino Acids

Branched chain amino acids (BCAAs), including leucine, valine, and isoleucine, are three major essential amino acids that play key roles in protein synthesis and energy metabolism. BCAAs function as activators of the mTOR signaling pathway (Jiang et al. 2023; Vanweert et al. 2022; Wolfson et al. 2016; Zhenyukh et al. 2017). As mTOR signaling is a central pathway of nutrient sensing and aging (Liu and Sabatini 2020), it is, therefore, reasonable to postulate that BCAA restriction may be related to longevity.

In mice, BCAA deprivation for 7 days improved insulin sensitivity (Xiao et al. 2014), and lifelong restriction of BCAA increased both healthspan and lifespan in a sex‐specific manner (Richardson et al. 2021). However, it was also reported that BCAA supplementation extends chronological lifespan in yeast and increases survival of mice by promoting mitochondrial biogenesis (D'Antona et al. 2010). In *C. elegans* , RNAi of branched‐chain amino acid transferase‐1 (*bcat‐1*) gene, which leads to BCAA accumulation, results in lifespan extension (Mansfeld et al. 2015). Activation of mTOR in *C. elegans* neurons due to a peripheral BCAA signal promotes lifespan through a cell‐non‐autonomous mechanism, which seems to conflict with our understanding that TOR inhibition extends lifespan. Dietary supplementation of BCAAs also extends worm lifespan, consistent with findings in mice (Mansfeld et al. 2015). In contrast, another study indicated that healthy aging might be associated with low BCAA intake (Solon‐Biet et al. 2014). Furthermore, BCAA promotes oxidative stress and inflammation in circulating blood cells and endothelial cells (Zhenyukh et al. 2017; Zhenyukh et al. 2018). In addition, BCAA accumulation drives SASP in fruit flies and mice (Liang et al. 2024). Elevated BCAA levels are also reported to lead to obesity, insulin resistance, and lifespan reduction (Solon‐Biet et al. 2019). A metabolomics‐based study of the relationship between blood BCAA levels and age in Chinese adults found that, among the three BCAAs, L‐Leu and L‐Ile were negatively associated with age, while L‐Val was upregulated in the elderly but showed no statistical correlation with age (Pan et al. 2023). These differential changes may be partly due to differences in their degradation pathways. These findings suggest that the roles of BCAAs in aging remain controversial, and such uncertainty may be due to the complexities of BCAA metabolic regulation and tissue‐specific effects. In addition, the poor concordance between dietary BCAA intake and circulating BCAA levels, as well as the influence of diseases or diet, may partly explain why associations between dietary BCAA and aging are inconsistent (Yao et al. 2023). Understanding the impact of BCAA on aging requires consideration of specific conditions, such as tissue type and life stage.

## 12 Vitamins

Vitamins are essential micronutrients that play diverse roles in physiology. While many are obtained from the diet, some can be endogenously produced. For example, Vitamin D_3_ (VD_3_) is a lipophilic secosteroid that functions as a hormone and is unique among vitamins because it is synthesized in skin cells (Santa et al. 2024). It is produced from the cholesterol precursor 7‐dehydrocholesterol through ultraviolet radiation, whereas the capacity of the epidermis to generate VD_3_ declines with age (MacLaughlin and Holick 1985). In *C. elegans* , exposure to 1000 μg/mL VD_3_ significantly increased lifespan by up to 39%, with partial dependence on the DAF‐12 nuclear receptor, which is homologous to the vitamin D receptor in humans (Messing et al. 2013). Another study reported that VD_3_ extends worm lifespan and promotes protein homeostasis through stress response regulators SKN‐1, IRE‐1, and XBP‐1 (Mark et al. 2016). A more recent study confirmed that VD_3_ significantly prolonged lifespan and healthspan (Huggins and Farris 2023). Strikingly, VD_3_ rescued the shortened lifespan of *nhr‐8* mutants, contrary to the expectation that its effects would require this human vitamin D receptor ortholog. In addition, VD_3_ upregulated genes linked to innate immunity and xenobiotic metabolism, with a significant enrichment for SKN‐1 (Huggins and Farris 2023). In rodents, VD_3_ has shown anti‐aging effects in specific tissues. In a D‐galactose‐induced aging rat model, VD_3_ supplementation significantly increased testicular germ cell proliferation and decreased apoptosis (Jeremy et al. 2019). Similarly, in aged rats, treatment of 42 and 420 IU/kg VD_3_ for 21 days reversed spatial memory deficits in 6‐ to 22‐month‐old animals (Bellettini‐Santos et al. 2023). VD_3_ also modulates both pro‐ and anti‐inflammatory cytokines mainly in the frontal cortex, indicating an immunomodulatory effect on aging (Bellettini‐Santos et al. 2023). However, human studies are less conclusive. A 16‐week randomized clinical trial in 70 vitamin D‐insufficient overweight African Americans found that 4000 IU VD_3_ daily slowed Horvath epigenetic aging by 1.85 years compared to placebo (Chen et al. 2019). However, a large‐scale, 5‐year randomized ancillary study in generally healthy older adults reported that 2000 IU VD_3_ daily had no effect on the change or incidence of frailty (Orkaby et al. 2022). Another 3‐year randomized controlled trial DO‐HEALTH reported only a small increase in lumbar spine and total hip areal bone mineral density with 2000 IU VD_3_ daily, but the effect sizes were minimal and the clinical relevance remains uncertain (Kistler‐Fischbacher et al. 2024). Thus, routine VD_3_ supplementation shows only modest physiological benefits in humans, and future work can focus on dosing for healthy aging and determine whether certain subgroups benefit more.

Vitamin C (VC), also named ascorbic acid, is a key water‐soluble antioxidant and cofactor. Unlike most mammals, humans lack the enzyme gulonolactone oxidase (GULO) and therefore cannot synthesize VC (Drouin et al. 2011). Studies on the effect of VC on aging have yielded mixed results. In an early review of 14 studies across worms, flies, and rodents, VC supplementation was associated with inconsistent effects on lifespan, ranging from extension to no benefit or even reduction (Pallauf et al. 2013). These inconsistent outcomes may attribute to heterogeneity in experimental conditions or species differences in endogenous VC synthesis. In the *Gulo* ^*−*/*−*^ mouse model, which lacks Gulo and mimics the scenario in humans, VC intake modulated metabolic profiles, decreased hepatic endoplasmic reticulum stress markers, and promoted longevity (Aumailley et al. 2016). Sun et al. recently discovered a CHIT1 (a secreted mammalian chitinase)‐high, neurotoxic microglial state in aged primate spinal cord that can trigger motor‐neuron senescence. The authors further showed that VC reversed CHIT1‐driven senescence and ameliorated motor neuron aging in aged monkeys (Sun et al. 2023). Taken together, overall evidence for VC supplements extending healthy lifespan is still weak and inconsistent; future research should conduct randomized trials across diverse populations.

Vitamin B_12_ (VB_12_) is a water‐soluble micronutrient that serves as a cofactor for two enzymes, methionine synthase and L‐methylmalonyl–coenzyme A mutase (Stabler 2013). Unlike VD_3_ or VC, VB_12_ is not synthesized by animal cells but is produced only by microorganisms, so animals must obtain it from diet or from microbial sources (Stabler 2013). VB_12_ deficiency is more common in older adults and is associated with several hallmarks of aging, including increased DNA damage, mitochondrial dysfunction, and epigenetic dysregulation (Simonenko et al. 2024). In humans, a meta‐analysis and a clinical trial both demonstrated that folic acid plus VB_12_ supplementation increased global DNA methylation, but evidence for consistent reductions in DNA methylation age is lacking and the effects on epigenetic age vary with sex and methylenetetrahydrofolate reductase genotype (Amenyah et al. 2020; Sae‐Lee et al. 2018). However, in a randomized trial, Obeid et al. found that participants supplemented with calcium + vitamin D (VD) + B‐vitamins (VB_6_, VB_9_, VB_12_) for 1 year showed higher ASPA methylation but lower PDE4C methylation compared with the calcium + VD group, suggesting a contradictory effect of B‐vitamins on epigenetic aging (Obeid et al. 2018). These discrepancies may be due to heterogeneity of interventions and the use of different methylation measurements. Importantly, all of these trials test VB_12_ in combination with other compounds, so the results should not be attributed to VB_12_ alone. Future work should therefore employ randomized trials that test VB_12_ individually.

## 13 Trehalose

Trehalose is a non‐reducing glucose disaccharide widely found in many bacteria, fungi, plants, and invertebrates, where it serves as a stress protectant (Zecic and Braeckman 2020). It is mainly synthesized from glucose‐6‐phosphate through a two‐step process involving trehalose‐6‐phosphate synthase (TPS) enzyme, which synthesizes trehalose‐6‐phosphate, followed by dephosphorylation of trehalose‐6‐phosphate to trehalose by trehalose‐6‐phosphate phosphatase (TPP) (Chen et al. 2022). However, mammals lack the enzymatic pathway to produce trehalose endogenously (Zecic and Braeckman 2020). The *C. elegans* expresses two TPS genes, *tps‐1* and *tps‐2*, and they are downstream targets of the IIS. TPS gene expression and trehalose levels are highly upregulated in dauers and the long‐lived *daf‐2* mutants, indicating their potential role in longevity and survival of extreme environments (Zecic and Braeckman 2020). Indeed, trehalose treatment extends the lifespan, healthspan, and reproductive span in worms (Honda et al. 2010). The lifespan‐extending effect of trehalose is abolished in *daf‐2* mutant, and RNAi of *tps‐1* or *tps‐2* also shortened the longevity of *daf‐2* mutant. Metabolic studies also show that diverting excess glucose into trehalose by inhibiting glycogen synthase *gsy‐1* markedly extends worm lifespan and healthspan, and this benefit requires the FOXO/DAF‐16 transcription factor and autophagy (Seo et al. 2018). These findings indicate trehalose‐mediated longevity requires IIS, and the benefits of reduced IIS is also, at least in part, dependent on the function of trehalose. However, evidence is mixed, as a recent report showed that even though *daf‐2* worms accumulate > 5‐fold trehalose, ablating trehalose synthesis did not shorten their lifespan (Rasulova et al. 2021), suggesting trehalose may support stress resilience more than directly driving longevity.

In other model animals, the effects of trehalose are also complex. In *Drosophila*, high dietary trehalose actually shortens female lifespan (Xu et al. 2023). In yeast, boosting trehalose metabolism by suppressing the HDA histone deacetylase increases stress resistance and replicative lifespan, and the effect is conserved in *C. elegans* and *Drosophila* (Yu et al. 2021). Likewise, deleting TOR/S6K pathway genes (*tor1* or *sch9*) in *S. cerevisiae* promotes a metabolic shift that uses acetic acid to accumulate trehalose and extends lifespan (Hu et al. 2014). Conversely, fission yeast shows no correlation between trehalose levels and lifespan. In mammals, the absence of trehalose synthesis and glyoxylate‐cycle genes means these metabolic anti‐aging mechanisms may not be fully conserved (Zecic and Braeckman 2020). Although cells do not make trehalose, exogenous trehalose can activate autophagy and improve mitochondrial quality in aging tissues. In mouse models of D‐galactose–induced reproductive aging, trehalose administration attenuated testicular aging by reducing germ‐cell apoptosis and restoring autophagic and mitochondrial quality control pathways, and it mitigated ovarian aging by decreasing granulosa cell death and increasing markers of autophagy and mitophagy such as LC3‐II, PINK1, and Parkin (Xi et al. 2024; Xi et al. 2025). These findings highlight that, although trehalose metabolism is involved in evolutionarily conserved mechanisms, its effect on aging may differ among species. The disparities likely reflect differences in experimental conditions, species‐specific metabolic differences, and dosage. Further work is needed to dissect the precise effects of trehalose metabolism on aging, to determine whether synthetic analogs or diet formulations might safely harness these pathways in mammals and to resolve these apparent inconsistencies.

## 14 Spermidine

Spermidine is an endogenous and evolutionarily conserved polyamine found across species, from bacteria to humans (Minois et al. 2011; Pegg 2009). It is synthesized intracellularly from putrescine via decarboxylated S‐adenosylmethionine (dcSAM) and can also be obtained from the diet (Bardocz et al. 1995; Seckute et al. 2011). Spermidine levels decline with age in mammals (Scalabrino and Ferioli 1984), and its supplementation has been extensively linked to longevity and health benefits. For example, in *C. elegans* , spermidine prevents neurodegeneration and improves behavioral outcomes via PINK1‐PDR1‐dependent mitophagy (Yang et al. 2020). In *Drosophila*, spermidine increases mitochondrial respiratory capacity and cognition in an autophagy‐dependent manner (Schroeder et al. 2021). The cross‐species geroprotective activity of spermidine was demonstrated by Eisenberg et al., who reported that spermidine supplementation markedly extends lifespan in yeast, *Drosophila* and *C. elegans* , and that it enhances survival of cultured human immune cells while reducing oxidative stress in aged mice. Spermidine promotes histone H3 deacetylation and autophagy, with autophagy required for the longevity benefit (Eisenberg et al. 2009).

In rodents, spermidine supplementation extends lifespan and yields organ‐protective effects including cardioprotection with preserved diastolic function dependent on cardiac autophagy (Eisenberg et al. 2016), reduced liver fibrosis and hepatocellular carcinoma incidence via MAP1S‐mediated autophagy (Yue et al. 2017), improved metabolic parameters and gut barrier integrity in diet‐induced obesity (Ma et al. 2020), cognitive benefits tied to increased eIF5A hypusination and mitochondrial function (Schroeder et al. 2021), and rejuvenates aged oocyte quality and improves fertility through enhanced mitophagy and mitochondrial function in aged mice (Zhang et al. 2023). A multispecies study found that fasting or CR elevates endogenous spermidine, and spermidine mediates benefits of fasting or CR by inducing autophagy and eIF5A hypusination (Bardocz et al. 1995). By contrast, a recent *Drosophila* study showed that protein restriction and exogenous spermidine each improve lifespan and brain function but act largely through distinct mechanisms, with spermidine effective regardless of dietary protein (Liang et al. 2025). This suggests that spermidine is not merely a universal downstream effector for all forms of DR interventions but may function as a critical hub for specific nutritional cues like fasting or CR, while acting in parallel to other interventions like protein restriction.

Clinical trials on spermidine show promising but complex results. Several studies correlate higher dietary or supplemental spermidine with positive outcomes like cognition and lower long‐term mortality, and several randomized trials are underway testing hypertension, heart failure with preserved ejection fraction (HFpEF), and vaccine responses (Guarente et al. 2024). By contrast, a population study found higher plasma spermidine associated with markers of advanced brain aging (Wortha et al. 2023). These discrepancies may be due to a discordance between intake and circulating spermidine levels, as polyamine catabolism can be upregulated in some disease states (Guarente et al. 2024; Wortha et al. 2023). Short‐term supplementation often fails to raise circulating spermidine, and sustained supplementation over many months may be required to produce measurable increases in blood spermidine levels (Soda 2022; Soda et al. 2021). Moreover, blood–brain barrier permeability to spermidine remains uncertain and may be increased in pathological conditions, which could contribute to inconsistent effects of spermidine treatment on brain aging (Wortha et al. 2023). Future randomized trials should include plasma and cerebrospinal fluid measurements together with validated assessments of blood–brain barrier permeability to determine whether spermidine supplementation yields meaningful peripheral and central exposure.

## 15 Lipid‐Related Metabolites

Ceramides are sphingolipid metabolites produced by de novo synthesis in the endoplasmic reticulum and by hydrolysis of membrane sphingomyelin by sphingomyelinases (Hannun and Obeid 2018). At low intracellular concentrations, ceramides support proliferation and survival, whereas at high concentrations, they promote cellular dysfunction and apoptosis (Cutler and Mattson 2001). In yeast, perturbing ceramide metabolism alters lifespan. For example, deletion of the C4‐hydroxylase SUR2 causes loss of phytoceramides, accumulation of dihydroceramides, impaired mitochondrial function, and reduced chronological lifespan (Deng et al. 2025). Likewise, a high‐sugar diet raises yeast ceramides and shortens chronological lifespan (Schmiedhofer et al. 2025). In multiple model organisms and humans, ceramides also accumulate with age and impair metabolic homeostasis. Targeted lipidomics showed that ceramide levels rise in aging *C. elegans* , mice, and humans, and inhibiting de novo ceramide synthesis restored proteostasis and mitochondrial function and extended worm lifespan (Lima et al. 2023). In mammals, an evolutionarily conserved GTPase GIMAP5 was found to limit pathological accumulation of long‐chain ceramides and thus modulates longevity‐related phenotypes (Park et al. 2024). Interventions that lower tissue ceramides produce metabolic benefits in rodents. Adipocyte‐specific overexpression of fibroblast growth factor 21 (FGF21) in adult mice fed a high‐fat diet lowered lipotoxic ceramides in visceral adipose tissue, improved systemic insulin sensitivity, and extended healthspan and lifespan (Gliniak et al. 2025). In humans, elevated plasma ceramides associate with insulin resistance and higher risk of type 2 diabetes, non‐alcoholic fatty liver disease, chronic kidney disease, and major adverse cardiovascular events (Fretts et al. 2020; Hilvo et al. 2020; Meeusen et al. 2018; Vasile et al. 2021). Bariatric surgery can reduce plasma ceramides and improve insulin sensitivity (Huang et al. 2011). Chronic exercise and some lipid‐lowering therapies have been linked to reductions in specific ceramide species in metabolically impaired participants (Hilvo et al. 2020; Reidy et al. 2020). However, randomized trials that test whether targeted lowering of ceramides has a direct impact on aging‐related health outcomes in humans are still lacking. In addition, although most studies position ceramides as negative regulators of metabolic health and as candidate drivers of accelerated aging, beneficial effects have also been reported. For instance, a human ceramide mixture (HC123) applied to skin fibroblasts in vitro stimulated transforming growth factor‐beta (TGF‐β) and fibroblast growth factor 2 (FGF2) signaling and markedly increased collagen and fibrillin production, implying potential dermal anti‐aging effects (Sugahara et al. 2022). Moreover, effects of ceramides on metabolism depend on acyl‐chain length and localization (Turpin‐Nolan and Bruning 2020). These findings emphasize the need for mechanistic clarity and indicate that translation requires species selective targeting, tissue specificity, and careful evaluation of beneficial versus harmful effects.

Ketone bodies, mainly β‐hydroxybutyrate (βHB), acetoacetate (AcAc), and acetone, are small molecules produced primarily by the liver from fatty acids during fasting, prolonged exercise, or very low carbohydrate intake to serve as an alternative energy source (Laffel 1999). Supplementation with βHB extends mean lifespan of *C. elegans* by about 20%. This lifespan extension is dependent on conserved longevity pathways, including DAF‐16/FOXO, SKN‐1/Nrf2, the sirtuin SIR‐2.1, and the AMPK subunit AAK‐2, and also involves the inhibition of histone deacetylases (Edwards et al. 2014). In mice, ketogenic diets initiated in mid‐life extended median lifespan by 13.6% and preserved cognitive or muscular function (Newman et al. 2017). CR is shown to increase circulating ketone bodies levels, and ketone bodies reproduce several other phenotypes of CR (Lin et al. 2015; Veech et al. 2017). It was therefore proposed that the benefits of CR are mediated at least in part by elevated ketone bodies, though this hypothesis requires further confirmation (Lin et al. 2015; Veech et al. 2017). In humans, supplementation with medium‐chain triglycerides, a nutritional source of ketone bodies, produces modest improvements or stabilization of cognitive performance in patients with AD or mild cognitive impairment (Juby et al. 2022; Reger et al. 2004). A recent work showed that deficiency in endogenous ketogenesis induced by whole‐body *Hmgcs2* deletion shortens lifespan in mice, and this effect is prevented by daily ketone body supplementation (Tomita et al. 2023). Conversely, constitutive feeding of ketone body‐containing diet from early life increases midlife mortality, even though it benefited aged or ApoE‐deficient mice. Furthermore, an ad libitum low carbohydrate ketogenic diet markedly increased mortality in the study. These findings suggest that endogenous ketogenesis is important for mammalian survival and that exogenous ketone interventions can be beneficial or harmful depending on administration method and the health status of the recipient, thereby warranting cautious and context‐specific translation to humans (Tomita et al. 2023).

## 16 Conclusions and Perspective

**FIGURE 1:**
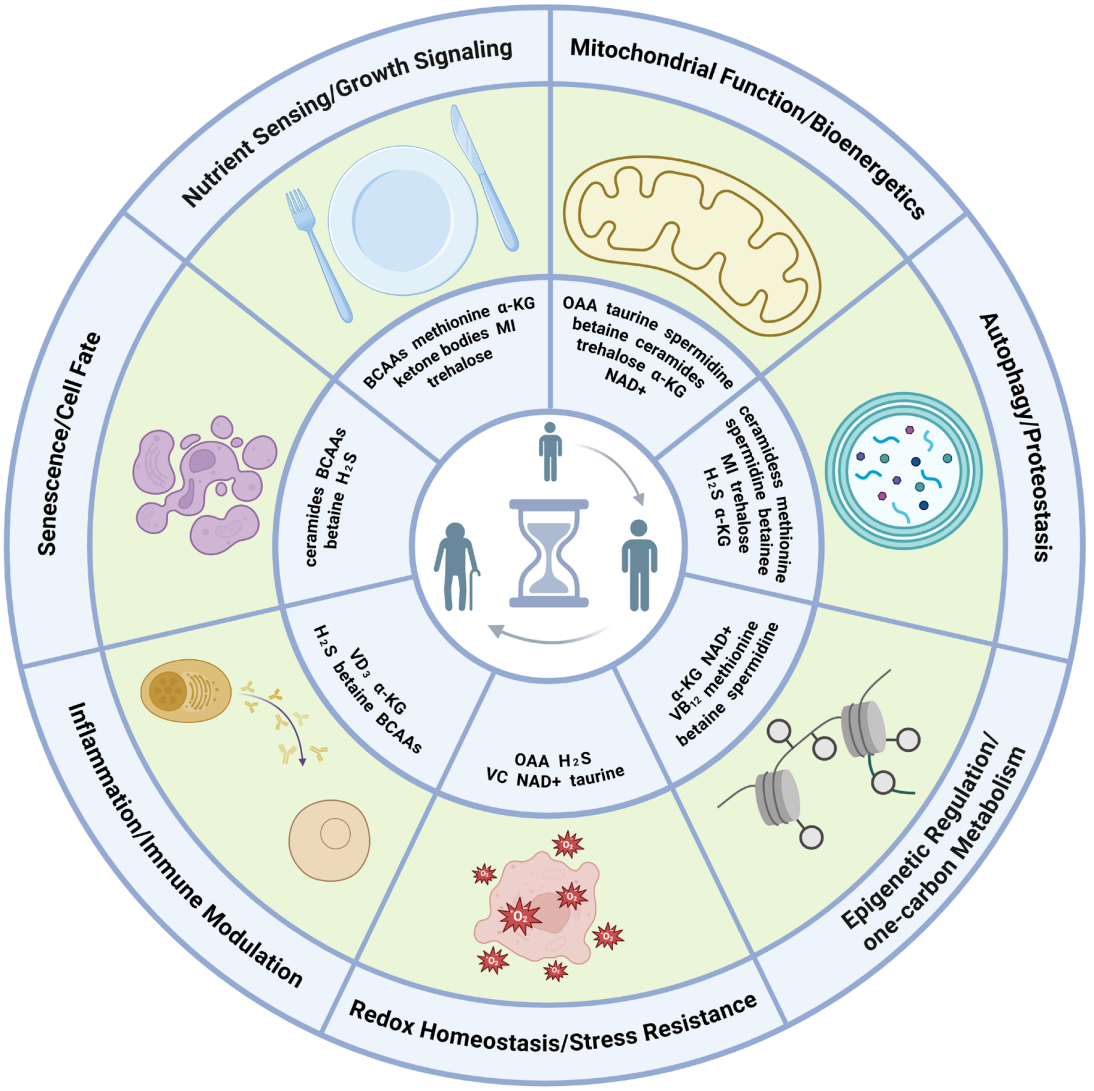
Endogenous metabolites and their mechanistic roles in conserved aging pathways. This schematic groups endogenous metabolites into seven aging‐related categories: Nutrient sensing/growth signaling, mitochondrial function/bioenergetics, autophagy/proteostasis, epigenetic regulation/one‐carbon metabolism, redox homeostasis/stress resistance, inflammation/immune modulation, and senescence/cell fate. Each sector covers two closely related but distinct mechanisms. Each metabolite is assigned to every primary mechanism for which supporting evidence is summarized in this review, and a metabolite within a given sector may act on one or both of the processes represented. Illustration created with BioRender.

Research on endogenous metabolites and longevity is a burgeoning field, yet significant translational gaps remain. While compelling evidence for lifespan extension has accumulated in invertebrates like worms and flies, supportive data in mammals are far fewer and typically limited to improvements in healthspan metrics rather than longevity itself, and evidence from randomized controlled human trials in anti‐aging effects is scarce. Key challenges include establishing optimal dosing, timing of intervention, and long‐term safety profiles before these preclinical findings can be translated into human applications. The mechanistic understanding of how endogenous metabolites influence longevity also remains incomplete. For instance, while methionine restriction extends lifespan across species, the precise molecular mechanisms underlying its effects on metabolism, cancer, and epigenetics continue to be a subject of debate (Ables et al. 2016). The effects of BCAAs on aging and insulin resistance are also controversial (Yao et al. 2023). In addition, while BCAAs may benefit some, they could have adverse effects in the elderly or those with inflammatory diseases, as they can promote a senescence‐associated inflammatory response (Liang et al. 2024). Other biological factors such as genetic background and sex can also modulate responses to dietary and metabolic interventions. A genetic and metabolomic study across 178 inbred *Drosophila* lines showed that genetic variation markedly alters baseline metabolite levels and the magnitude of lifespan extension produced by DR, suggesting that genetic background may determine who benefits from metabolic interventions (Jin et al. 2020). A recent study in mice found that lifelong restriction of dietary valine improved multiple healthspan measures in both sexes but extended median lifespan by approximately 23% only in males and not in females (Calubag et al. 2025). This heterogeneity, therefore, necessitates personalized, precision‐medicine approaches in the translation of these findings. Likewise, studies should explore tissue‐specific actions and the influence of metabolites on various physiological systems. Key findings on each metabolite's origin, anti‐aging effects, and mechanisms are summarized in Table **1**, and Figure **1** classifies the metabolites by the conserved aging‐related pathways they are associated with.

**TABLE 1:** Summary of lifespan‐extending endogenous metabolites. This table summarizes endogenous metabolites discussed in this review, with species of endogenous origin, evidence for anti‐aging effects in invertebrates and mammals, and clinical evidence.

Metabolite	Endogenous origin	Anti‐aging effects in invertebrates	Anti‐aging effects in mammals	Clinical evidence
Taurine	Synthesized endogenously in many vertebrates; present in diet	*C. elegans* : ↑ lifespan (Singh et al. 2023)	Mice: ↓photoaging (Yoshimura et al. 2021); ↓cognitive decline, ↑memory (El Idrissi et al. 2013); ↓senescence, ↑mitochondrial function (Singh et al. 2023)	Mixed results; taurine levels variable with age (Fernandez et al. 2025; Singh et al. 2023; Vincent Marcangeli et al. 2025)
Betaine	Synthesized from choline in plants, bacteria, and animals; abundant in dietary sources	*C. elegans* : ↑ lifespan via DAF‐16, p38‐MAPK, mTOR inhibition, autophagy (Lan et al. 2024)	Mice: ↑ muscle function, ↑ cognition, ↑ autophagy, mimics exercise via TBK1 inhibition (Chen, He, et al. 2024; Geng et al. 2025; Yang et al. 2024)	Observational/epidemiologic evidence mixed (Zawieja and Chmurzynska 2025); high‐dose betaine (4–6 g/day) associated with ↑LDL in some trials (Ashtary‐Larky et al. 2022); no RCT showing anti‐aging effects
α‐Ketoglutarate (α‐KG)	Universal TCA cycle intermediate	*C. elegans* : ↑ lifespan via ATP synthase inhibition, ↓ TOR, ↑ AMPK/FOXO (Chin et al. 2014); *Drosophila*: ↑ lifespan (Su et al. 2019)	Mice: ↑ lifespan and healthspan (Asadi Shahmirzadi et al. 2020); improves age‐related phenotypes (osteoporosis, cardiac dysfunction, oocyte aging) (An et al. 2021; H. Wang, Xu, Li, et al. 2023; Wang et al. 2020; Ye et al. 2024)	↓ DNA methylation age (Demidenko et al. 2021); Ca‐AKG trial registered (Sandalova et al. 2023)
Oxaloacetate (OAA)	Produced in TCA cycle in bacteria, plants, and animals	*C. elegans* : ↑ lifespan via AMPK activation and DAF‐16/FOXO; proposed NAD^+^/NADH shift (Williams et al. 2009); *Drosophila*: OAA levels correlate with lifespan (Harrison et al. 2022)	Mice: no lifespan extension in genetically heterogeneous UM‐HET3 mice (Strong et al. 2013); ↑ neuromuscular function in SOD1^G93A ALS mice but did not increase survival (Tungtur et al. 2021)	Safe in Alzheimer's trial, no cognitive benefit (Vidoni et al. 2021)
Hydrogen sulfide (H_2_S)	Endogenously produced in animals by CBS, CSE, and 3‐MST enzymes; also produced by some bacteria	*C. elegans* (H_2_S donors): ↑ lifespan extension via SIR‐2.1, ATF‐4 (Miller and Roth 2007; Statzer et al. 2022)	Mice (H_2_S donors): protects heart/kidney, ↓SASP (Lee et al. 2018; Zhang et al. 2021); AD models: Tau protection, ↑ cognition (Giovinazzo et al. 2021)	—
Myo‐Inositol (MI)	Synthesized from glucose‐6‐phosphate in many organisms; abundant in plant foods and mammalian tissues	*C. elegans* : ↑ lifespan via PTEN/DAF‐18 & PINK1 mitophagy (Shi et al. 2020); ↑ lifespan via PI3K/AKT/DAF‐16 (Yang et al. 2023)	Mice: ↑ cardiac function, ↓ROS/DNA damage (Mingyao et al. 2024); ↑ hippocampal MI linked to glial activation & cognitive decline (Ebert et al. 2021)	—
NAD^+^ (nicotinamide adenine dinucleotide)	Ubiquitous cofactor synthesized de novo from tryptophan or via salvage pathways from vitamin B3 (niacin) in bacteria, plants, and animals	Yeast/ *C. elegans* : boosting NAD^+^ (e.g., NR, NMN) ↑ Sir2/Sirtuin activity and ↑ lifespan (Verdin 2015; Yoshino et al. 2011)	Mice: NMN/NR supplementation ↑ metabolic and physiological functions and delays age‐related decline (Wang et al. 2016)	Short‐term NMN/NR trials increased blood NAD^+^ and reported modest functional improvements (Yi et al. 2023); larger RCTs show mixed results (Orr et al. 2024; Szarvas et al. 2025)
Methionine (Met)	Essential amino acid from diet; central to SAM‐mediated one‐carbon metabolism in bacteria, plants, and animals	Yeast: Methionine restriction (MetR) ↑ lifespan via autophagy and vacuolar acidification (Ruckenstuhl et al. 2014); *C. elegans/Drosophila*: ↑ lifespan with MetR or altered Met metabolism (Cabreiro et al. 2013; Kosakamoto et al. 2023; Liu et al. 2019)	Mice/Rats: MetR ↑ lifespan (Orentreich et al. 1993); ↓IGF‐1, ↑FGF21, AMPK activation (Ables et al. 2016; Lees et al. 2014)	—
Branched‐chain amino acids (BCAA: Leu, Ile, Val)	Essential amino acids from diet, produced by microbes/plants; found across animals	Yeast: ↑ chronological lifespan (D'Antona et al. 2010); *C. elegans* : BCAT‐1 RNAi or BCAA supplementation ↑ lifespan (Mansfeld et al. 2015); *Drosophila*: ↑ BCAA induces SASP (Liang et al. 2024)	Mice: lifelong BCAA restriction ↑ lifespan in a sex‐specific manner (Richardson et al. 2021); short‐term BCAA deprivation ↑ insulin sensitivity (Xiao et al. 2014); chronic high BCAA intake links to metabolic dysfunction (Solon‐Biet et al. 2019); ↑BCAA induces SASP (Liang et al. 2024)	—
Vitamin D_3_ (VD_3_)	Synthesized in vertebrate skin from 7‐dehydrocholesterol upon UV exposure; also obtained from diet	*C. elegans* : ↑ lifespan and ↑ proteostasis via SKN‐1/IRE‐1/XBP‐1 and partly requires DAF‐12 (Huggins and Farris 2023; Mark et al. 2016; Messing et al. 2013)	Rats: ↑ cognition/testicular health (Bellettini‐Santos et al. 2023; Jeremy et al. 2019)	Mixed human data: 4000 IU/day slowed epigenetic aging in one 16‐week trial (Chen et al. 2019), but large trials found no clear benefit for frailty (Orkaby et al. 2022) or only small bone‐density gains (Kistler‐Fischbacher et al. 2024)
Vitamin C (VC; ascorbic acid)	Most mammals synthesize VC; humans lack GULO and depend on dietary VC	Mixed outcomes across invertebrate (Pallauf et al. 2013)	Mice (Gulo−/−): VC supplementation ↑ lifespan, ↓ ER stress (Aumailley et al. 2016); Monkeys: reverses neurotoxic microglial senescence (Sun et al. 2023)	—
Vitamin B_12_ (VB_12_; cobalamin)	Synthesized by bacteria and archaea; animals acquire VB_12_ from diet or gut microbes	*C. elegans* : dietary VB_12_ deficiency ↓ fecundity and ↓ lifespan; dietary VB_12_ status modulates development, fertility, and lifespan (Bito et al. 2013; Bito et al. 2017; Nair et al. 2022)	Mammals: VB_12_ deficiency is associated with DNA damage, mitochondrial dysfunction, and epigenetic dysregulation (Simonenko et al. 2024)	Folic acid + VB_12_ supplementation increased global DNA methylation (Amenyah et al. 2020; Sae‐Lee et al. 2018); effects on epigenetic aging inconsistent (Obeid et al. 2018)
Trehalose	Synthesized by bacteria, fungi, plants and invertebrates, not synthesized by mammals	*C. elegans* : ↑ lifespan via DAF‐16 & autophagy (Honda et al. 2010; Seo et al. 2018); *Drosophila*: high trehalose ↓ lifespan (Xu et al. 2023); Yeast: ↑ trehalose metabolism ↑ lifespan (Hu et al. 2014; Yu et al. 2021)	Mice (D‐galactose‐induced reproductive aging): exogenous trehalose ↑ autophagy, mitigates ovarian/testicular aging (Xi et al. 2024; Xi et al. 2025)	—
Spermidine	Synthesized from putrescine via decarboxylated S‐adenosylmethionine; also obtained from diet	Yeast/*Drosophila*/ *C. elegans* : ↑ lifespan (Eisenberg et al. 2009); *C. elegans* : prevents neurodegeneration and ↑ behavior via PINK1‐PDR1 dependent mitophagy (Yang et al. 2020); *Drosophila*: ↑ mitochondrial respiratory capacity and cognition in an autophagy‐dependent manner (Schroeder et al. 2021)	Mice: ↑ lifespan and organ protection: preserved diastolic function (Eisenberg et al. 2016), reduced liver fibrosis and hepatocellular carcinoma (Yue et al. 2017), improved metabolic parameters and gut barrier in diet‐induced obese mice (Ma et al. 2020), improved cognition linked to increased eIF5A hypusination and mitochondrial function (Schroeder et al. 2021), rejuvenated aged oocyte quality via enhanced mitophagy (Zhang et al. 2023)	Observational signals and small interventions are mixed; several randomized trials are ongoing; short‐term supplementation often fails to raise circulating spermidine (Soda 2022; Soda et al. 2021)
Ceramides	Produced by de novo synthesis in the endoplasmic reticulum or by sphingomyelin hydrolysis	Yeast: perturbing sphingolipid/ceramide metabolism alters lifespan (Deng et al. 2025); *C. elegans* : inhibiting de novo ceramide synthesis restores proteostasis and ↑ lifespan (Lima et al. 2023)	Mice: adipose‐specific FGF21 overexpression in high‐fat diet induced obese mice ↓ ceramide levels in visceral adipose tissue, ↑ insulin sensitivity, ↑ healthspan and ↑ lifespan in some models (Gliniak et al. 2025)	Higher plasma ceramides associate with higher risk of type 2 diabetes, non‐alcoholic fatty liver disease, chronic kidney disease, and major adverse cardiovascular events (Fretts et al. 2020; Hilvo et al. 2020; Meeusen et al. 2018; Vasile et al. 2021); Trials testing targeted ceramide lowering for anti‐aging outcomes are lacking
Ketone bodies	Produced in liver during fasting, prolonged exercise or very low carbohydrate intake; main species: β‐hydroxybutyrate (βHB), acetoacetate (AcAc), acetone	*C. elegans* : βHB supplementation ↑ lifespan via DAF‐16/FOXO SKN‐1/Nrf2 SIR‐2.1 and AAK‐2/AMPK and involves HDAC inhibition (Edwards et al. 2014)	Mice: midlife ketogenic diet ↑ lifespan, preserves cognition and muscle function (Newman et al. 2017); endogenous ketogenesis deficiency shortens lifespan and can be rescued by daily ketone supplementation; early‐life ketogenic diet may increase midlife mortality in some conditions (Tomita et al. 2023)	Medium‐chain triglycerides supplementation produces modest improvements or stabilization of cognitive performance in patients with AD or mild cognitive impairment (Juby et al. 2022; Reger et al. 2004)

Overall, endogenous metabolites hold significant translational potential due to their safety profile, as many are naturally occurring in foods. Some metabolites, such as betaine and BCAAs, are already utilized clinically for other indications (Holecek 2017; Wilcken et al. 1983). Although humans cannot synthesize certain metabolites such as VC, VB_12_, and trehalose, they are endogenous to other species and can modulate conserved pathways that regulate aging, for example, antioxidant defenses, one carbon metabolism, and autophagy (Simonenko et al. 2024; Xi et al. 2025). In addition, some of these metabolites become relatively depleted with age; for example, VB_12_ shows a high prevalence of insufficiency in older adults, resulting in disrupted cellular homeostasis (Simonenko et al. 2024). Therefore, targeted supplementation of these metabolites to restore physiological levels may counteract age‐related deficits that drive functional decline. Nevertheless, altering metabolic processes may lead to unintended consequences. For instance, while BCAAs are beneficial in some contexts, excessive levels may exacerbate metabolic conditions, such as insulin resistance or metabolic syndrome (Yao et al. 2023). Rigorous, controlled studies are essential to further investigate these effects. In conclusion, focusing on endogenous metabolic regulators offers a promising complementary approach to traditional drug and genomic strategies in aging research. Addressing existing gaps and standardizing classifications will enable researchers to more effectively harness these small molecules to promote healthy aging and longevity.

## Author Contributions

Yizhou Jiang: original draft preparation. Jing‐Dong J. Han: review and editing. All authors agreed and approved this article version to be submitted in this journal.

## Funding

This work was supported by Hainan Medical University (RZ2500001786 and RZ2300005972), the National Natural Science Foundation of China (32088101, 92374207, 32330017 and 82361148130), and the Beijing Natural Science Foundation (IS23077 and L254002).

## Conflicts of Interest

The authors declare no conflicts of interest.

## Data Availability

Data sharing not applicable to this article as no datasets were generated or analyzed during the current study.
